# Contrast-enhanced ultrasound thyroid imaging reporting and data system outperforms K-TIRADS in diagnostic accuracy and reduces unnecessary fine-needle aspirations for thyroid nodules

**DOI:** 10.3389/fendo.2026.1866681

**Published:** 2026-07-21

**Authors:** Guanchun Chen, Jingliang Ruan, Rongbin Liu, Youmei Tu, Zishen Ou, Zihao Zhong, Fuying Tao, Hesong Ye, Baoming Luo

**Affiliations:** 1 Ultrasound Department, Houjie Hospital of Dongguan (Emergency Hospital of Dongguan), Dongguan, China; 2 Department of Ultrasound, Sun Yat-sen Memorial Hospital, Sun Yat-sen University, Guangzhou, China

**Keywords:** CEUS TI-RADS, contrast-enhanced ultrasound, diagnostic accuracy, fine-needle aspiration, K-TIRADS, thyroid nodule, unnecessary biopsy rate

## Abstract

**Background:**

Thyroid nodules are frequently detected on ultrasonography, yet only a small proportion are malignant. The Korean Thyroid Imaging Reporting and Data System (K-TIRADS) is widely used for risk stratification; however, its reliance solely on grayscale ultrasonographic features may result in suboptimal diagnostic performance and unnecessary fine-needle aspirations (FNAs). Contrast-enhanced ultrasound (CEUS) provides real-time visualization of tumor microvascular perfusion patterns, which may complement conventional ultrasound findings.

**Objective:**

This study aimed to evaluate a contrast-enhanced ultrasound-based Thyroid Imaging Reporting and Data System (CEUS TI-RADS) compared with K-TIRADS for differentiating benign from malignant thyroid nodules and to assess its ability to reduce unnecessary FNAs.

**Methods:**

A total of 175 thyroid nodules in patients who underwent FNA and/or surgical resection between January 2022 and December 2023 were retrospectively enrolled. Each nodule was classified according to both K-TIRADS and CEUS TI-RADS criteria, with pathological results serving as the reference standard. Receiver operating characteristic (ROC) curve analysis was performed to compare the diagnostic efficacy of the two systems. Sensitivity, specificity, accuracy, positive predictive value (PPV), negative predictive value (NPV), and unnecessary biopsy rate (UBR) were calculated. Multivariate logistic regression was used to identify independent predictors of malignancy.

**Results:**

Among 175 nodules, 84 were malignant and 91 were benign. CEUS TI-RADS demonstrated significantly higher diagnostic performance than K-TIRADS, with an area under the ROC curve (AUC) of 0.893 versus 0.804 (P < 0.05). CEUS TI-RADS achieved a substantially lower UBR at 18.6% compared with K-TIRADS 2016 at 44.8% and with K-TIRADS2021 at 44.8% (P < 0.01). Multivariate logistic regression identified hypoechoic echogenicity (P = 0.036), irregular or lobulated margins (P = 0.003), punctate echogenic foci (P < 0.01), and hypoenhancement (P = 0.007) as independent predictors of malignancy.

**Conclusion:**

CEUS TI-RADS offers superior diagnostic accuracy for differentiating benign from malignant thyroid nodules compared with K-TIRADS and effectively reduces the rate of unnecessary fine-needle aspirations. These findings support the clinical utility of incorporating CEUS-based features into thyroid nodule risk stratification.

## Introduction

Thyroid nodules represent one of the most common endocrine disorders, with a continuously increasing global prevalence (1). High-resolution ultrasound detects thyroid nodules in 20%–76% of the general population, among which only 7%–15% are malignant (2). Widespread neck ultrasound leads to massive incidental nodule detection, bringing great challenges to distinguish benign and malignant lesions and avoid unnecessary invasive procedures (3).

Three mainstream global thyroid risk stratification frameworks (ACR TI-RADS, C-TIRADS, K-TIRADS) have distinct applicable populations and inherent defects, which we systematically compared here. ACR TI-RADS was built on Western patient cohorts, mismatching Asian nodule morphology (4). C-TIRADS separately scores comet-tail artifacts and integrates cervical lymph node status into grading, causing poor reproducibility (5). In contrast, K-TIRADS 2016/2021 was developed from Asian data with internationally consistent scoring logic, making it the optimal control for our research (6, 7). The Korean Thyroid Imaging Reporting and Data System (K-TIRADS) is one of the most extensively utilized risk stratification models worldwide and has substantially standardized the ultrasonographic diagnostic workflow for thyroid nodules. We selected K-TIRADS (2016 and 2021 versions) rather than C-TIRADS or ACR TI-RADS as the control for three key reasons. First, our study cohort consisted of Asian patients, and K-TIRADS was developed based on Asian population data, which matches our enrolled population’s demographic features, unlike ACR TI-RADS established primarily for Western populations. Second, while C-TIRADS targets Asian populations as well, it regards comet-tail artifact as an independent grading variable for thyroid nodules, inconsistent with the grading rules of most mainstream international TI-RADS systems. According to ACR TI-RADS criteria, tiny comet-tail artifacts are classified as punctate echogenic foci and scored 3 within hypoechoic lesions; importantly, comet-tail artifact alone is insufficient to define benign nodules. Third, C-TIRADS incorporates cervical lymph node status into nodule risk scoring, whereas ultrasonic evaluation of cervical lymph nodes has limited diagnostic accuracy and poor interobserver consistency in clinical practice, potentially impairing the reproducibility of C-TIRADS classification (5, 8). Therefore, K-TIRADS was adopted as the comparator to achieve objective and internationally comparable performance verification of CEUS TI-RADS (9). However, similar to other grayscale ultrasound-based TIRADS systems, K-TIRADS inevitably shows considerable interobserver variability and overlapping ultrasonic manifestations between benign and malignant nodules. More importantly, it only depends on static morphological features without integrating intranodular microcirculation perfusion information, which easily causes over-stratification or under-stratification, especially for category 4 indeterminate nodules, leading to a high unnecessary FNA rate and potential missed diagnosis of early malignancy.

Contrast-enhanced ultrasound (CEUS) enables real-time visualization of tumor microvascular perfusion and reflects blood supply differences between pathological and normal tissues (10). Published meta-analyses reported the sensitivity, specificity, positive predictive value, and negative predictive value of CEUS in thyroid malignancy diagnosis as approximately 85%, 82%, 83%, and 85%, respectively (11). Nevertheless, CEUS feature heterogeneity leads to inconsistent diagnostic criteria and controversial clinical application. Ruan et al. established a CEUS TI-RADS combining grayscale and CEUS features, but it lacks large-scale multicenter external validation (9). The present study aimed to validate the diagnostic performance of CEUS TI-RADS for thyroid nodules and to compare it with K-TIRADS.

## Materials and methods

### Study design and ethical approval

This retrospective study was approved by the Ethics Committee of Dongguan Emergency Hospital (Approval No. 2022-047). Written informed consent was obtained before CEUS, FNA or surgical procedures. The study complied with the Declaration of Helsinki.

### Patients

Consecutive eligible thyroid nodules from January 2022 to December 2023 were enrolled via consecutive sampling method.

Inclusion criteria were: (a) availability of postoperative histopathological or FNA cytological results, and (b) maximum nodule diameter ≥ 5 mm. Exclusion criteria were: (a) prior thyroid surgery, chemotherapy, radiotherapy, thermal ablation or ¹³¹I therapy; (b) nodules classified as Bethesda I, III, or IV without definite pathological conclusions; and (c) nodules without definite pathological follow-up or surgical results. Bethesda V nodules were not excluded because all these highly suspicious nodules received surgical resection and obtained definite postoperative pathological results, which enriched the composition of malignant cases and ensured diagnostic reliability. All malignant nodules in this study were confirmed by postoperative histopathology as the gold standard. Benign nodules were restricted to Bethesda II lesions with ≥12 months stable ultrasound follow-up; relevant study proved the false-negative risk of this standard was controlled below 3.2%, reducing cytology-derived bias (12).

### Ultrasound examination

All patients were placed in the supine position with a neck pillow to fully expose the anterior cervical region for standard scanning. For patients with multiple thyroid nodules, each eligible nodule was numbered independently and analyzed as a separate research unit.

Conventional and CEUS examinations were performed using a Samsung RS80A ultrasound system (Samsung Medison, Seoul, South Korea), with a 3–12 MHz linear array transducer for conventional ultrasound and a 2–9 MHz transducer for CEUS. The contrast agent used was sulfur hexafluoride microbubbles (SonoVue, Bracco, Milan, Italy).

Grayscale and color Doppler images were acquired on standard transverse and longitudinal planes that fully displayed nodule maximum size, boundary, internal echo and calcification features, defined as the most representative planes to reduce subjective image selection bias. For CEUS, the transducer was fixed at the optimal nodule plane. A 1.2 mL contrast agent bolus was injected intravenously, followed by 5 mL normal saline flush. Mechanical index was set to 0.08. CEUS dynamic observation lasted at least 120 seconds, and the entire perfusion process was stored by cine-loop recording for subsequent offline analysis.

### Image analysis

**Figure 1:**
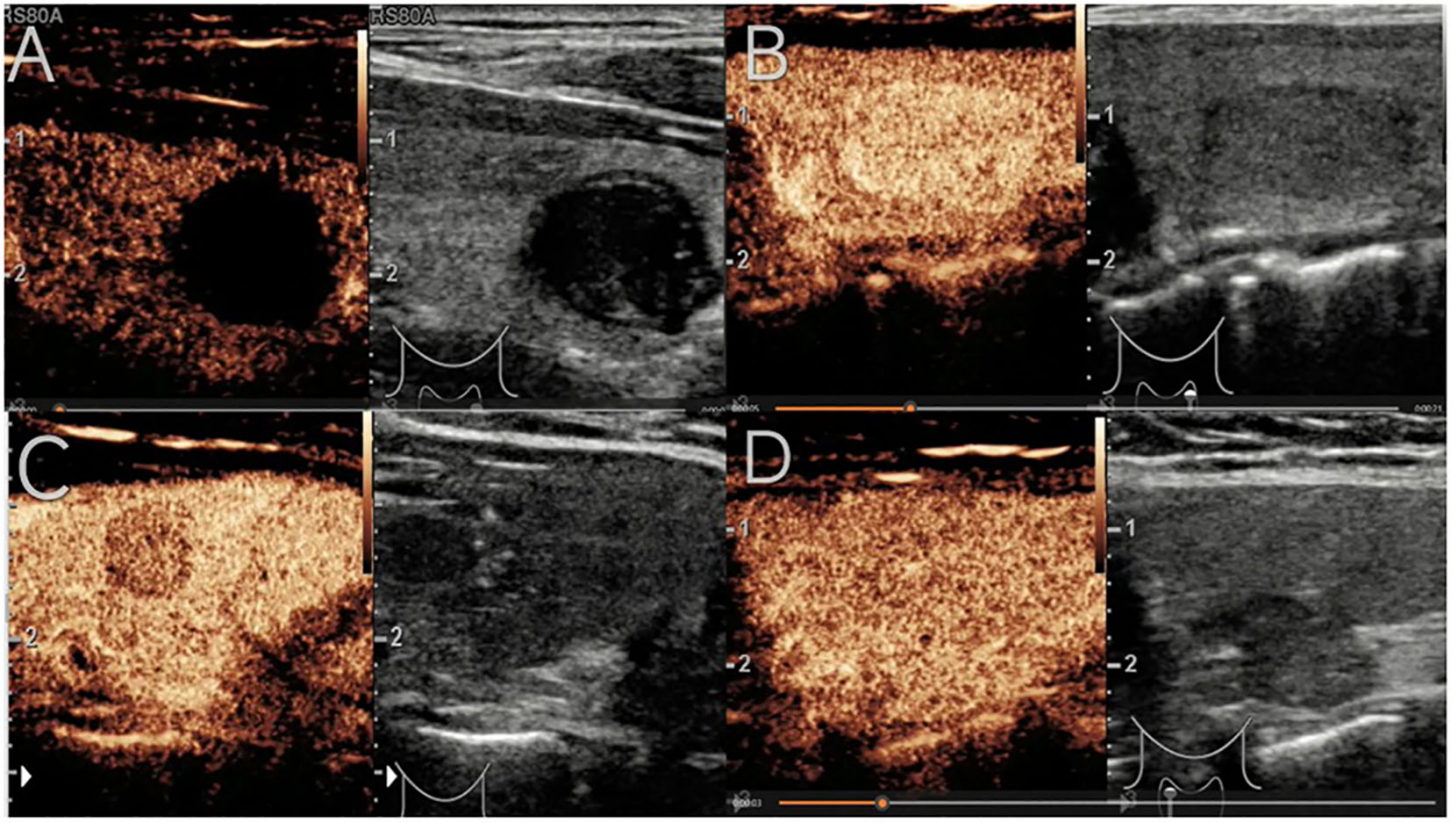
Contrast-enhanced ultrasound (CEUS) image (left) and conventional ultrasound image (right). **(A)** Non-enhancing nodule with benign FNA pathology; non-enhancement is a highly specific benign marker. **(B)** Homogeneous hyperenhancement, benign nodule. **(C)** Hypoenhancement confirmed papillary thyroid carcinoma (PTC) by surgery. **(D)** Isoenhancement, benign nodule. Abbreviations: CEUS, contrast-enhanced ultrasound; FNA, fine-needle aspiration; PTC, papillary thyroid carcinoma.

**Figure 2:**
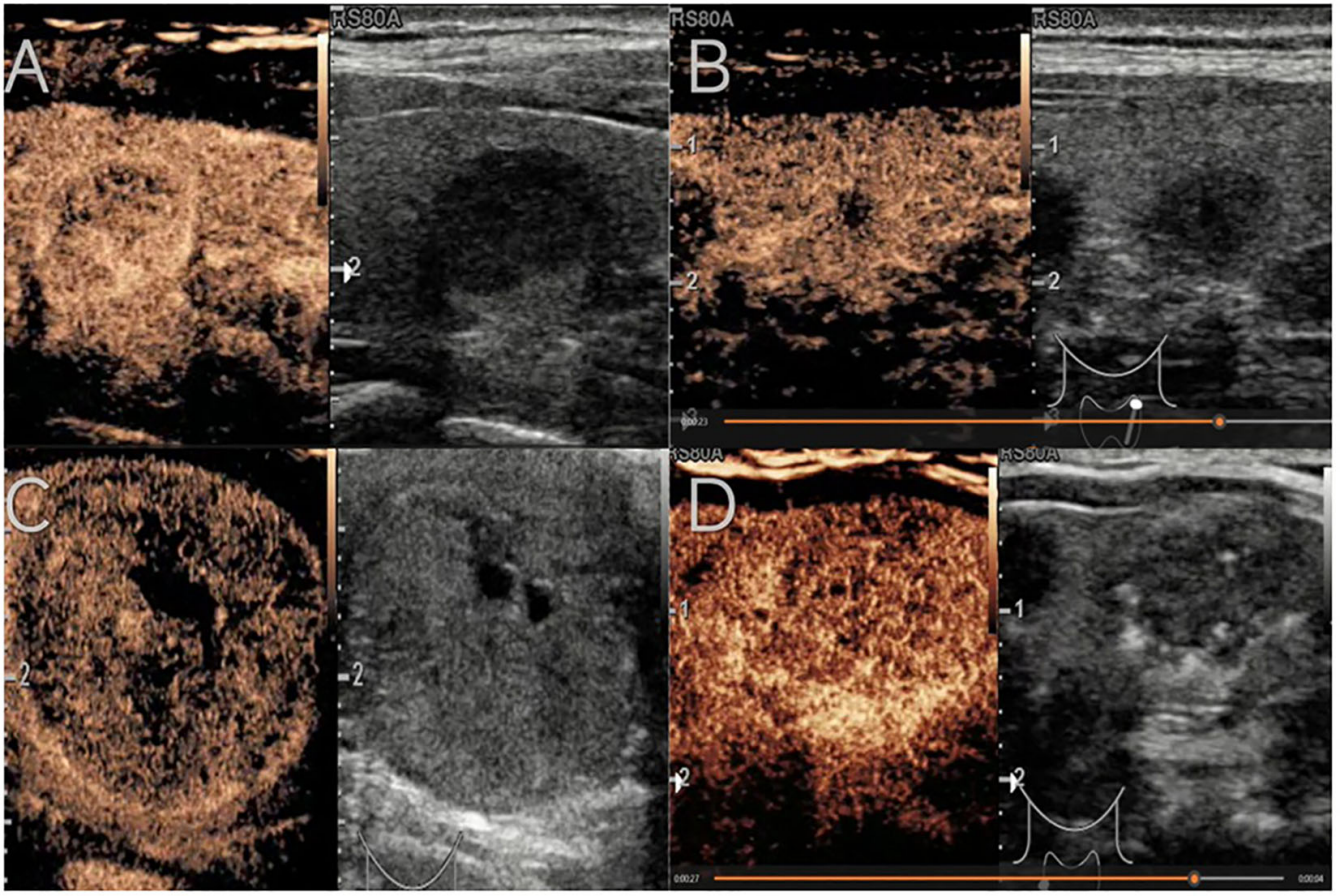
CEUS image (left) and conventional ultrasound image (right). **(A)** Complete peripheral ring enhancement, benign lesion on FNA. **(B)** Absent ring enhancement, surgical PTC. **(C)** Cystic nodule with focal perfusion defect, benign. **(D)** Solid hypoperfused nodule, PTC.

Image interpretation was performed by two attending radiologists with more than 8 years of thyroid ultrasound diagnostic experience, who were fully blinded to pathological results and clinical information. Conventional ultrasound features included nodule composition, echogenicity, shape, margins, echogenic foci, and extrathyroidal extension. CEUS features included contrast arrival time, enhancement direction, enhancement pattern, peak intensity, ring enhancement, and CEUS nodule composition (**Figures 1**, **2**). If a solid nodule on conventional ultrasound showed no enhancement throughout CEUS, it was scored 0 and classified into corresponding TI-RADS category.

### Statistical analysis

Statistical analyses were performed using SPSS version 27.0 (IBM Corp., Armonk, NY, USA). Quantitative data were expressed as mean ± standard deviation. The chi-square test was used to compare the qualitative ultrasound features between benign and malignant nodules. ROC curve analysis was applied, and optimal cut-off values were determined by the maximum Youden index. Sensitivity, specificity, accuracy, positive predictive value (PPV), negative predictive value (NPV), and unnecessary biopsy rate (UBR) were calculated. Multivariate logistic regression was used to identify independent predictors of malignancy. *Post-hoc* statistical power and sample size validation was performed based on ROC-AUC comparison. Assuming baseline AUC 0.80 for K-TIRADS, expected AUC increase 0.09, α = 0.05, power = 0.80, the minimal required sample size was 156 nodules. Our 175 enrolled nodules exceeded this threshold, confirming adequate statistical power for subsequent analyses. For multivariate logistic regression, clinical epidemiological standards require at least 10 malignant outcome events per independent variable; our model contained 8 imaging predictors and included 84 malignant nodules, fully meeting the power requirement for stable regression fitting (13). Cohen’s squared weighted Kappa was used to assess inter-observer consistency of ordinal CEUS TI-RADS and K-TIRADS classifications between the two attending radiologists. Discrepant grading results reached consensus via mutual discussion for subsequent diagnostic statistical analysis. A professional biostatistician audited all statistical design and calculation processes, with audit records available upon editorial request. A P value of < 0.05 was considered statistically significant.

## Results

### Patient characteristics

A total of 175 nodules (84 malignant, 91 benign) from 32 male and 143 female patients were enrolled, aged 18–80 years (mean 43.9 ± 11.4 years). Nodule maximum diameter ranged 5–54 mm (mean 12.9 ± 9.5 mm). The mean age of benign nodule patients was 46 ± 14 years, significantly older than 42 ± 11 years in malignant group (P = 0.018). No significant differences were found in gender and nodule size (P > 0.05).

Significant differences existed in echogenicity, shape, margins, echogenic foci and extrathyroidal extension between benign and malignant nodules (all P < 0.05). All CEUS features also showed significant intergroup differences (all P < 0.05). Baseline characteristics are shown in **Table 1**.

**Table 1:** Demographic and patient characteristics of participants with thyroid nodules.

Characteristic	Benign nodules (n=91)	Malignant nodules (n=84)	P value
Number of nodules	91	84	
Mean age (years)	46 ± 14	42 ± 11	0.018
Gender			0.521
Male	15	17	
Female	76	67	
Nodule size			0.286
≤1 cm	36	45	
>1 cm	55	39	
Echogenicity			<0.001
Hyperechoic/Isoechoic	42	8	
Hypoechoic	48	71	
Markedly hypoechoic	1	5	
Shape			0.001
Wider-than-tall	72	47	
Taller-than-wide	19	37	
Margins			<0.001
Smooth	57	20	
Ill-defined	31	26	
Irregular	1	15	
Lobulated	2	23	
Echogenic foci			<0.001
None	51	26	
Large comet-tail artifacts	9	3	
Coarse calcifications	20	7	
Rim calcifications	4	2	
Punctate echogenic foci	7	48	
Extrathyroidal extension			0.003
Present	0	6	
Absent	91	78	
CEUS: Enhancement direction			<0.001
Scattered	71	20	
Centripetal/Centrifugal	20	64	
CEUS: Peak intensity			<0.001
Isoenhancement	37	14	
Hyperenhancement	35	10	
Hypoenhancement	15	60	
Non-enhancement	4	0	
CEUS: Ring enhancement			<0.001
Absent	59	76	
Present	32	8	
CEUS: Nodule composition			0.004
Non-solid	24	8	
Solid	67	76	

### Inter-observer agreement for TI-RADS classification

Weighted Kappa analysis revealed moderate inter-observer agreement for both grading systems. For CEUS TI-RADS, the weighted κ = 0.554 (95%CI: 0.398–0.686); for K-TIRADS, the weighted κ = 0.545 (95%CI: 0.364–0.690). All inconsistent classifications between two readers were resolved by consensus to determine the final TI-RADS category used for subsequent diagnostic performance calculation.

### Comparison of diagnostic performance between CEUS TI-RADS and K-TIRADS

**Figure 3:**
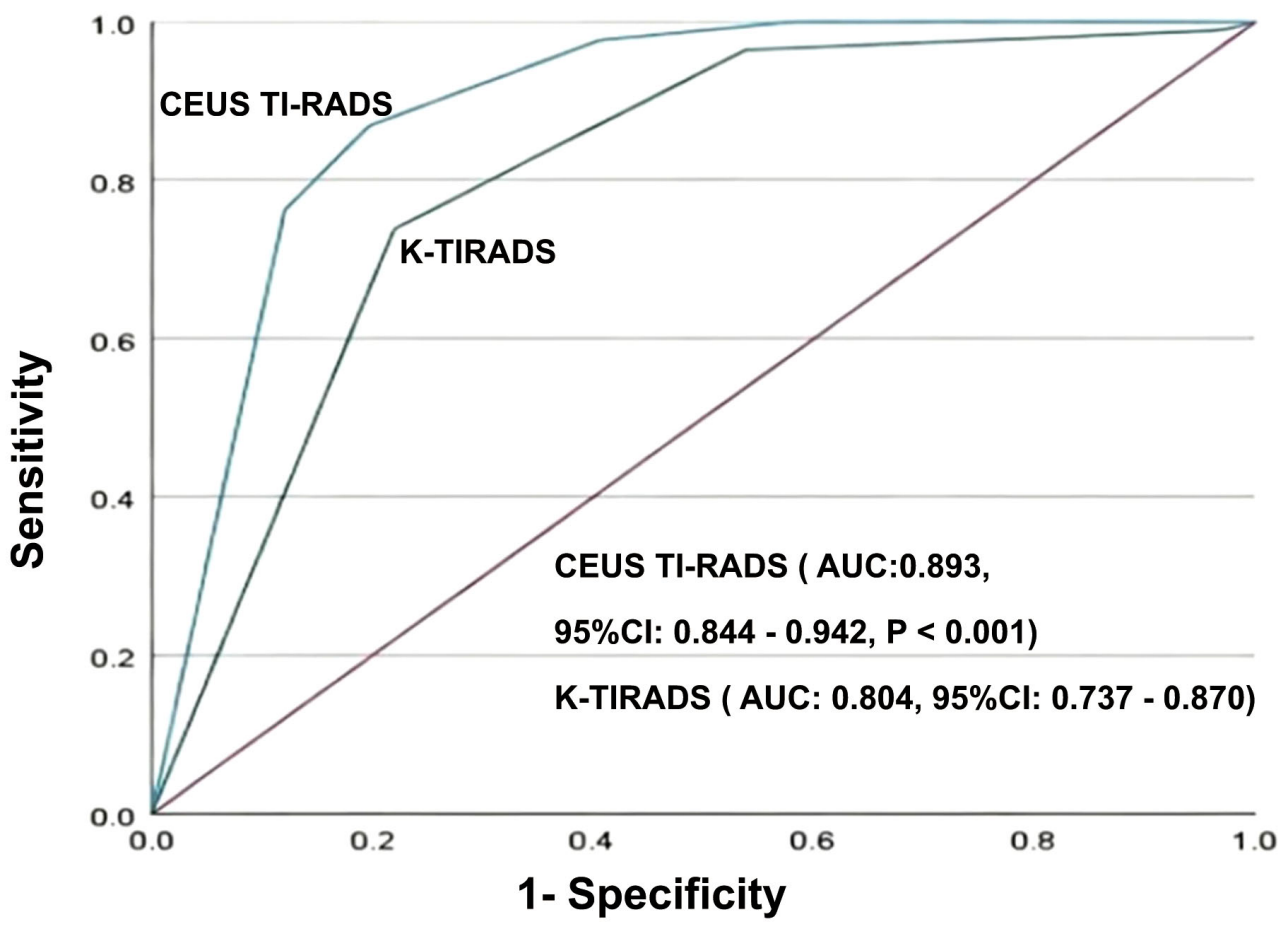
Receiver operating characteristic (ROC) curves comparing the diagnostic performance of K-TIRADS and CEUS TI-RADS for predicting thyroid nodule malignancy. CEUS TI-RADS AUC = 0.893 (95%CI 0.844–0.942), K-TIRADS AUC = 0.804 (95%CI 0.737–0.870), inter-group P<0.001.

CEUS TI-RADS showed significantly higher sensitivity, specificity, accuracy, PPV, NPV and AUC than K-TIRADS (all P < 0.05). AUC was 0.893 (95% CI: 0.844–0.942) vs. 0.804 (95% CI: 0.737–0.870) (**Table 2** and **Figure 3**). Cut-off values were 5.5 for CEUS TI-RADS and 4.5 for K-TIRADS.

**Table 2:** Comparison of diagnostic performance between K-TIRADS and CEUS TI-RADS for thyroid nodule classification.

Parameter	K-TIRADS	CEUS TI-RADS
Sensitivity	76.3%(71/93)	86.9%(73/84)
Specificity	75.6%(62/82)	80.2%(73/91)
PPV	78.0%(71/91)	80.2%(73/91)
NPV	73.8%(62/84)	86.9%(73/84)
Accuracy	76.0%(133/175)	83.4%(146/175)
AUC	0.804	0.893
P value	< 0.001	< 0.001
95% CI	0.737–0.870	0.844–0.942

PPV, positive predictive value; NPV, negative predictive value; AUC, area under the receiver operating characteristic curve.

CEUS TI-RADS yielded a significantly higher overall malignant biopsy rate of 81.4% (35/43) and a much lower overall unnecessary biopsy rate (UBR) of 18.6% (8/43), versus the 55.2% malignant biopsy rate and 44.8% UBR (74/134, 60/134) observed for both K-TIRADS 2016 and K-TIRADS 2021 (P < 0.01). Tiered risk subgroup analysis showed consistent advantages of CEUS TI-RADS across all risk levels. For low-risk nodules, UBRs were 92% (K-TIRADS 2016) and 90% (K-TIRADS 2021), compared with 66.7% for mildly suspicious CEUS TI-RADS nodules. For intermediate-risk lesions, UBRs were 63.0% (K-TIRADS 2016), 54.5% (K-TIRADS 2021), and 33.3% for moderately suspicious CEUS TI-RADS nodules. For high-risk nodules, CEUS TI-RADS highly suspicious lesions achieved a UBR of 0%, far lower than the 24.4% UBR of high-suspicion nodules in both K-TIRADS versions (**Table 3**).

**Table 3:** Comparison of unnecessary biopsy rate between K-TIRADS and CEUS TI-RADS.

Categories	Malignant biopsy rate	Unnecessary biopsy rate	P value
K-TIRADS2016	55.2% (74/134)	44.8% (60/134)	0.004
Low Suspicion	8% (2/25)	92% (23/25)	0.159
Intermediate Suspicion	37% (10/27)	63% (17/27)	0.172
High Suspicion	75.6% (62/82)	24.4% (20/82)	0.003
K-TIRADS2021	55.2% (74/134)	44.8% (60/134)	0.004
Low Suspicion	10% (2/20)	90% (18/20)	0.218
Intermediate Suspicion	45.5% (10/22)	54.5% (12/22)	0.410
High Suspicion	75.6% (62/82)	24.4% (20/82)	0.003
CEUS TI-RADS	81.4% (35/43)	18.6% (8/43)	N/A
Mildly Suspicious	33.3% (2/6)	66.7% (4/6)	N/A
Moderately Suspicious	66.7% (8/12)	33.3% (4/12)	N/A
Highly Suspicious	100% (25/25)	0 (0/25)	N/A

### Logistic regression analysis of ultrasound and CEUS features

Univariate analysis showed that most conventional and CEUS features differed significantly between benign and malignant nodules. Multivariate logistic regression identified hypoechoic echogenicity (P = 0.036), irregular/lobulated margins (P = 0.003), punctate echogenic foci (P < 0.01), and hypoenhancement (P = 0.007) as independent malignant predictors (**Table 4**). Detailed clinical interpretation of each odds ratio was added in **Table 3** footnote to quantify relative malignant risk.

**Table 4:** Multivariate logistic regression analysis of independent predictors of malignant thyroid nodules.

Ultrasound feature	B	SE	P value	Exp(B)	95% CI
Taller-than-wide	0.733	0.583	0.209	2.081	0.664–6.520
Hypoechoic	−1.456	0.693	0.036	0.233	0.060–0.908
Irregular/lobulated	−2.160	0.736	0.003	0.115	0.027–0.488
Punctate echogenic foci	−2.343	0.589	< 0.01	0.096	0.030–0.304
Enhancement direction	−0.646	0.617	0.295	0.524	0.156–1.757
Hypoenhancement	−1.901	0.700	0.007	0.149	0.038–0.590
Absence of ring enhancement	−0.434	0.746	0.561	0.648	0.150–2.798
CEUS composition	−1.039	0.743	0.162	0.354	0.082–1.518

Exp(B)=odds ratio; lower Exp(B) means higher malignant risk. Hypoechoic Exp(B)=0.233 (76.7% lower benign risk); irregular margins Exp(B)=0.115 (88.5% lower benign risk); punctate foci Exp(B)=0.096; hypoenhancement Exp(B)=0.149 (85.1% lower benign risk).

## Discussion

The present study systematically validated the diagnostic performance of CEUS TI-RADS and compared its efficacy with both the 2016 and 2021 versions of K-TIRADS. Our results demonstrated that CEUS TI-RADS achieved a higher AUC value and obviously reduced the unnecessary biopsy rate (UBR) compared with both K-TIRADS versions. The superior diagnostic performance of CEUS TI-RADS is mainly attributed to the addition of tumor microvascular perfusion information, which compensates for the inherent deficiency of conventional grayscale ultrasound that only relies on morphological morphology for risk stratification. Cross-system comparison of ACR TI-RADS, C-TIRADS and K-TIRADS further proved all grayscale-only grading frameworks lack perfusion indicators and lead to elevated UBR, while CEUS TI-RADS solves this core defect.

In our enrolled cohort, both the UBR of K-TIRADS 2016 and K-TIRADS 2021 were 44.8%. For category 4 indeterminate nodules, the UBR remained as high as 63.0% for K-TIRADS 2016 and 54.5% for K-TIRADS. These findings are consistent with previous validation studies, which reported a UBR of approximately 60.9% for K-TIRADS category 4 nodules and 48.6% when adopting a 1.0 cm biopsy threshold (14). It is evident that the 2021 revised criteria indeed optimized the stratification strategy for small nodules and category 4 lesions, leading to a moderate reduction in unnecessary biopsy rate compared with the 2016 version. Nevertheless, both versions of K-TIRADS still showed markedly higher UBR than CEUS TI-RADS. Grade-stratified UBR results illustrated CEUS TI-RADS optimized risk stratification for Category 3, 4 and 5 simultaneously, not merely intermediate-risk lesions.

The clinical advantage of CEUS TI-RADS was particularly prominent in the management of K-TIRADS category 4 nodules, which are the most common and challenging indeterminate lesions in daily clinical practice. Conventional grayscale ultrasound features often overlap considerably between benign and malignant category 4 nodules, making accurate stratification difficult. By revealing real-time microcirculatory perfusion characteristics, CEUS provides additional independent diagnostic evidence: typical hypoenhancement and centripetal perfusion are strongly associated with malignancy, whereas ring enhancement shows high specificity for benign lesions (15, 16). In addition, CEUS exhibits unique diagnostic value for special nodule subtypes such as mummified thyroid nodules, which are easily misclassified by conventional ultrasound alone, and CEUS can effectively correct such misjudgment by identifying non-enhancement characteristics (17).

Compared with previously reported quantitative CEUS scoring models and complicated multimodal algorithms (18–20), the CEUS TI-RADS adopted in this study relies entirely on qualitative feature evaluation, with simple and time-saving operating procedures (average examination time within 5 min) (21, 22). It has low dependence on operator experience and is more suitable for popularization in primary hospitals and routine clinical workflow, which provides good application prospects for clinical transformation. However, pure qualitative evaluation brings inevitable subjective reading bias; quantitative CEUS perfusion parameters (time-to-peak, peak intensity) can supply objective digital thresholds to lower inter-reader discrepancy, and we plan to construct dual-modal scoring combining qualitative and quantitative indicators in follow-up research. Recent multicenter external validation studies of CEUS-based thyroid grading systems confirmed that integrating perfusion features universally reduces unnecessary biopsy rates in Asian cohorts (23, 24). Multimodal diagnostic evidence confirmed that combining CEUS with shear wave elastography further improves differential diagnosis efficiency for nodules coexisting with Hashimoto’s thyroiditis (25).

Several limitations of this study should be acknowledged. First, over 94% of malignant nodules were papillary thyroid carcinoma, which may limit the generalizability to other pathological subtypes such as follicular carcinoma. Follicular and medullary carcinoma usually present atypical perfusion patterns, which may reduce the diagnostic sensitivity of this system; future multicenter prospective cohorts will enrich rare malignant subtypes and conduct subgroup stratified analysis. Second, this was a single-center retrospective study, which inevitably carries selection bias and lacks multicenter external validation. Third, unified operational specifications and image quality evaluation criteria for CEUS still need further standardization and expert consensus. Fourth, the moderate inter-observer reproducibility of conventional and CEUS TI-RADS grading was consistent with real-world ultrasound practice, partially caused by subjective judgment differences for borderline indeterminate nodule features. Three core reasons caused moderate Kappa consistency: ambiguous borderline lesion features, pure qualitative perfusion evaluation, absent national CEUS unified consensus; future solutions include standardized multi-center reader training and AI-assisted perfusion feature recognition. In addition, benign diagnoses in the current study relied on stable long-term follow-up of Bethesda II FNA results instead of postoperative pathology alone, which is an unavoidable limitation of retrospective design. Although cytology-related false-negative risk was controlled below 3.5%, residual bias existed; future prospective research will only adopt surgical histopathology as the unique gold standard to eliminate this interference. Future prospective research will enroll only nodules with surgical histopathology as the reference standard to eliminate potential bias from FNA cytology results.

From the perspective of clinical translation, CEUS TI-RADS provides a promising optimization scheme for thyroid nodule risk stratification. For nodules classified as category 4 by K-TIRADS (either 2016 or 2021 criteria), routine supplementary CEUS examination is recommended to reduce unnecessary invasive biopsy. For nodules complicated with Hashimoto’s thyroiditis, the combination of CEUS and elastography may further improve diagnostic accuracy. Future research should enroll more pathological subtypes and adopt multicenter prospective designs to further verify the stability and generalizability of CEUS TI-RADS. In addition, combining artificial intelligence with CEUS image analysis is expected to further improve the objectivity and inter-observer consistency of thyroid nodule diagnosis.

## Conclusion

This study demonstrates that CEUS TI-RADS provides superior diagnostic accuracy for differentiating benign from malignant thyroid nodules compared with K-TIRADS and effectively reduces unnecessary fine-needle aspirations. These findings support the clinical utility of incorporating CEUS-based features into thyroid nodule risk stratification.

## Data Availability

The original contributions presented in the study are included in the article/supplementary material. Further inquiries can be directed to the corresponding author.
